# Feeding Preference Differentiation in Sympatric Willow Leaf Beetles Driven by Host Leaf Trait Divergence

**DOI:** 10.3390/ani16152395

**Published:** 2026-08-03

**Authors:** Lixuan Kou, Fan Cheng, Jinghang Lian, Zhiwei Liang, Sheng Zhang, Hao Wang, Di Zhang, Jiansheng Wang, Peisong Liu

**Affiliations:** 1 Henan Key Laboratory of Germplasm Innovation and Utilization of Eco-Economic Woody Plant, Pingdingshan University, Pingdingshan 467000, China; koulx2015@163.com (L.K.);; 2 College of Geography and Environmental Engineering, Pingdingshan University, Pingdingshan 467000, China; 3 College of Chemistry and Chemical Engineering, Pingdingshan University, Pingdingshan 467000, China

**Keywords:** leaf beetle, feeding preference, niche differentiation, morphological traits, alpine meadow

## Abstract

Insect herbivores often exhibit host preference and feeding differentiation underpinning fine-scale niche partitioning, yet their linkage to larval performance remains poorly understood. On the Qinghai–Tibet Plateau, two sympatric leaf beetles, *Chrysomela vigintipunctata alticola* (CV) and *Phratora vitellinae* (PV) (both Coleoptera: Chrysomelidae), co-occur on two willow species occupying distinct microhabitats: CV predominantly uses abundant *Salix atopantha* (SW), while PV specializes on rare *Salix wangiana* var. *tibetica* (LW) (both Malpighiales: Salicaceae). To disentangle intrinsic feeding response from natal-host induction, we combined dual-choice feeding bioassays with controlled natal-host rearing experiments using first-instar larvae originating from both hosts, and evaluated fitness consequences in terms of morphology and instar duration. PV larvae exhibited a consistent preference for LW, coupled with wider head capsules. In contrast, CV larvae showed no intrinsic preference; specifically, those from SW remained non-selective, while those from LW developed a delayed preference. CV larvae developed faster on nitrogen-rich SW, whereas LW contained higher salicylate esters. These results indicate that PV prioritizes growth and chemical defensive sequestration on LW, whereas CV prioritizes rapid development by utilizing the nutrient-rich SW. Our results reveal distinct resource-use patterns and life-history strategies that facilitate local co-occurrence of the two leaf beetles within the alpine meadow ecosystem.

## Simple Summary

On the Qinghai–Tibet Plateau, two leaf beetles—*Chrysomela vigintipunctata alticola* (CV) and *Phratora vitellinae* (PV)—share the same habitat but feed on different willow species. In the field, CV mainly feeds on the abundant small-leaf willow (SW), while PV prefers a rare, large-leaf willow (LW). We tested whether this pattern is driven by larval feeding preference and its fitness consequences. Through dual-choice larval feeding bioassays and controlled rearing experiments, we found that PV larvae possess a strong, intrinsic preference for LW, which supports their growth and chemical defense sequestration. In contrast, CV larvae lack an intrinsic preference but develop significantly faster on SW. This divergence in life-history trade-offs (defensive investment vs. accelerated development) facilitates resource partitioning and likely supports local coexistence under restrictive alpine growing conditions.

## 1. Introduction

Host selection and feeding preference are fundamental processes shaping insect–plant interactions, population dynamics, and community structure [1,2,3]. For herbivorous insects, selective feeding on specific host plants or tissues represents a key behavioral strategy linked to larval performance, which reduces interspecific competition, and promotes stable coexistence among sympatric species [4,5]. Unraveling the mechanisms that link feeding preference to larval performance is therefore critical for understanding species coexistence and community dynamics [6].

To minimize competitive pressure, closely related or sympatric herbivores frequently partition resources along multiple niche axes, including food quality, phenology, and spatial distribution [7,8,9,10]. Such niche partitioning is largely facilitated by variation in host plant traits. Willows (*Salix* spp.) (Malpighiales: Salicaceae) serve as dominant hosts for many leaf beetles and exhibit substantial variation in leaf size, texture, nutrient content, and defensive chemistry [11,12,13]. These diverse traits create distinct microhabitats and resource gradients, allowing closely related beetles to partition resources [14,15,16]. However, the mechanisms by which leaf trait divergence drives feeding preference differentiation among sympatric willow-feeding beetles, and how this differential host use subsequently shapes larval performance to mediate coexistence, remain poorly understood.

On the Qinghai–Tibet Plateau, two sympatric willow leaf beetles, *Chrysomela vigintipunctata alticola* Wang (CV) and *Phratora vitellinae* (L.) (PV) (Coleoptera: Chrysomelidae), co-occur on willow host plants but occupy distinct microhabitats. CV and PV feed solely on alpine willows native to the eastern Qinghai–Tibet Plateau. CV is an endemic high-altitude subspecies mainly found in habitats over 3200 m [17], while PV is widely distributed across the Palaearctic realm [18]. Dense local populations may cause partial defoliation and alter alpine meadow community structure [19,20]. Field observations show that CV occurs primarily on *Salix atopantha* C. K. Schneider (small-leaf willow: SW) [19], with only a small proportion found on *Salix wangiana* var. *tibetica* C. Wang & C.F. Fang (large-leaf willow: LW). In contrast, PV is predominantly found on LW, with occasional occurrence on SW. These two willow taxa are endemic alpine *Salix* shrubs confined to high-altitude meadows of the eastern Qinghai–Tibet Plateau, where abundant endemic willow species have evolved specialized adaptations to harsh alpine climate [21]. As dominant woody shrub components in alpine shrubby meadows, they serve as exclusive host resources for specialized willow-feeding leaf beetles under harsh climatic constraints. These two willows differ markedly not only in leaf morphology but also presumably in defensive and nutritional traits, which are key mediators of willow-leaf beetle interactions [12,13]. Despite these preliminary field patterns, quantitative evidence for feeding preference differentiation and its consequences for larval performance remains lacking. It remains unclear whether this observed host association patterns stem from active adult oviposition choice or mere density-dependent encounter rates. To distinguish between these mechanisms, we examined larval innate feeding preference and performance to uncover the mechanistic basis driving adult host selection—specifically, whether adults oviposit to match offspring preference and optimize fitness.

The Qinghai–Tibet Plateau, the highest and largest plateau in the world located in southwestern China, is characterized by short growing seasons, low temperatures, and strong ultraviolet radiation, which exert strong selective pressure on insect development and survival [22,23]. Alpine herbivores must therefore balance rapid development, nutrient acquisition, and anti-predator defense to complete their life cycles before winter [24,25]. For willow-feeding leaf beetles, interspecific variation in willow leaf size reflects not only resource quantity but also nutritional quality, defensive chemistry, and microclimatic suitability [1,26]. Although leaf size is widely recognized as a key plant functional trait, its role in mediating feeding preference and subsequent larval fitness remains understudied in alpine systems. Furthermore, under such extreme temporal constraints, whether adults consistently prioritize larval preference or are forced to compromise growth for rapid development remains unknown. Resolving these contrasting host-use mechanisms advances our knowledge of resource partitioning in alpine herbivore assemblages with short growing seasons.

This study aimed to quantify first-instar larval feeding preference and larval performance associated with divergent leaf traits, so as to infer the intrinsic drivers underlying adult host preference in natural populations. We conducted dual-choice feeding assays and natal-host rearing experiments to test the following hypotheses derived from the preference-performance hypothesis [27,28,29]: (1) CV larvae prefer SW whereas PV larvae prefer LW; (2) larvae reared on their preferred leaf type exhibit enhanced growth (greater body length and wider head capsules); and (3) host preference aligns with improved larval performance. By integrating behavioral, morphological, developmental, and chemical data, we elucidated the mechanisms driving resource partitioning and host-associated feeding differentiation in alpine leaf beetle communities.

## 2. Materials and Methods

### 2.1. Study Site

**Figure 1:**
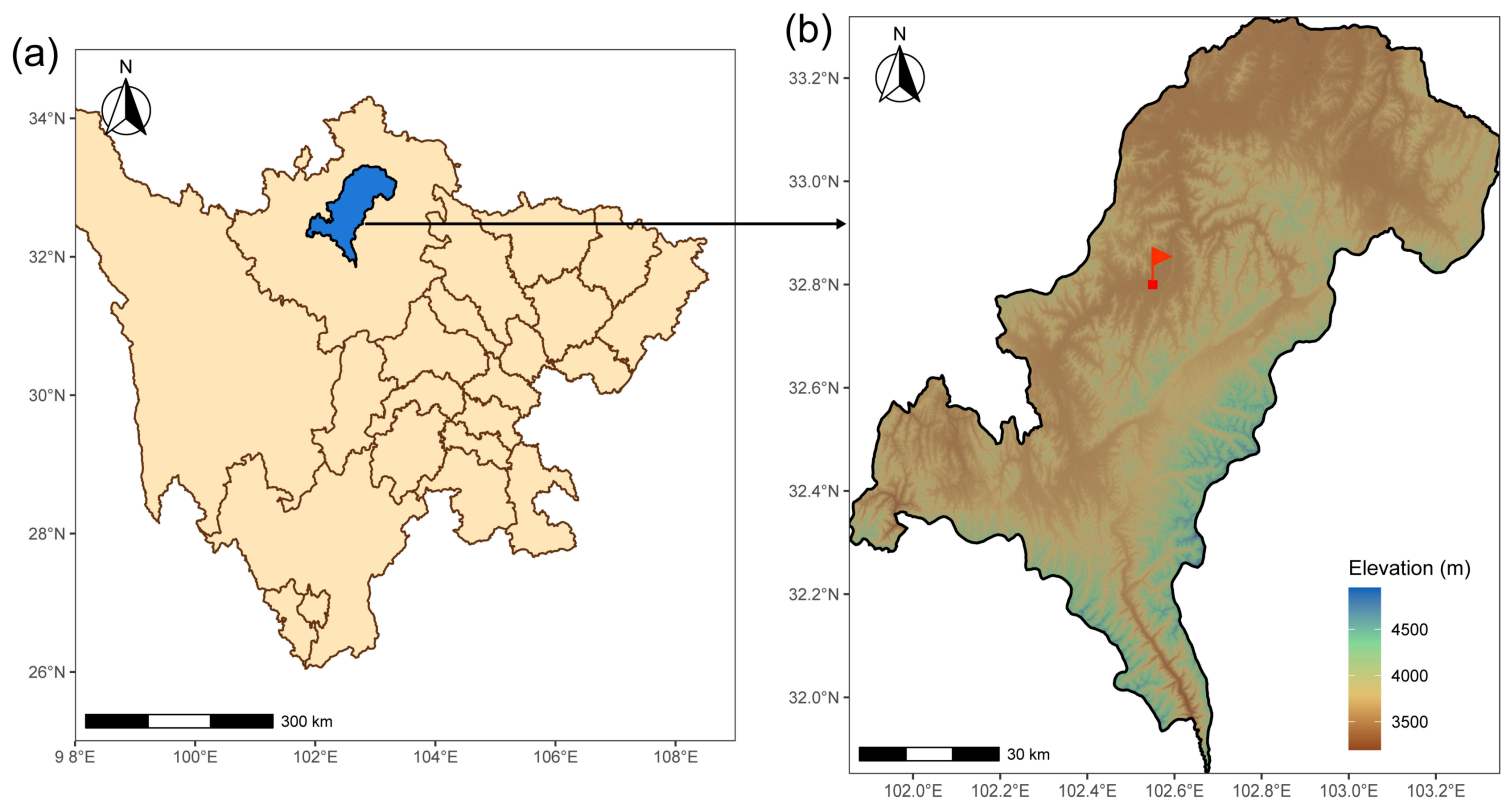
Geographic location of the study site in Hongyuan County, Sichuan Province, China. (**a**) Location of Hongyuan County within Sichuan Province; (**b**) Digital elevation model (DEM) of Hongyuan County, with the field experimental site denoted by a red flag. The colour bar displays elevation values. Brown hues represent elevations below 3500 m, blue hues indicate elevations above 4500 m, and intermediate gradient colours represent terrain between 3500 to 4500 m. Administrative boundary data for Sichuan Province and Hongyuan County were downloaded from the DataV platform (https://datav.aliyun.com, accessed 1 May 2026). The 30 m resolution GDEMV3 digital elevation model (DEM) data were obtained from the Geospatial Data Cloud (https://www.gscloud.cn/sources/index?pid=1&rootid=1, accessed 1 May 2026). All map layers were processed and assembled using R 4.5.3 [32].

This study was conducted in Hongyuan County, Sichuan Province, China, on the eastern part of the Qinghai–Tibet Plateau (32°48′ N, 102°33′ E, ~3500 m a.s.l.; Figure 1). The region has a typical alpine meadow climate, with a long, cold winter (October to April) and a short, cool summer (July to August) [30]. The minimum winter temperature can drop below −25 °C, with frozen soil depth exceeding 30 cm, and all aboveground plant parts senesce during winter. The mean annual temperature is 1.7 °C, and annual precipitation is 754 mm (data from Hongyuan County Meteorological Station, 5.5 km from the study site) [31]. Summer is marked by large diurnal temperature fluctuations (15–20 °C) and intense ultraviolet radiation.

The vegetation is dominated by herbaceous alpine meadow species [33], with scattered willow shrubs as key host plants for leaf beetles. Willow plants in this region are slow-growing and highly adapted to low temperatures, with leaf expansion and beetle larval hatching tightly synchronized with the short summer growing period. This phenological synchrony makes the system ideal for testing feeding preference and performance linked to leaf traits. Two willow types coexist here: *Salix atopantha* (small-leaf willow: SW); *Salix wangiana* var. *tibetica* (large-leaf willow: LW). Due to the short growing season, these willows exhibit strong phenological overlap, producing new leaves in early May. Leaf beetles oviposit in early June, and larvae feed intensively throughout the summer [19].

### 2.2. Leaf Size Measurements

At least 30 fresh, fully expanded, undamaged leaves were randomly collected from the middle canopy at four cardinal directions (north, south, east, west) across at least 6 individual shrubs of each willow species (SW and LW). Each leaf was photographed with a ruler for scale, and the leaf area was calculated using ImageJ software (Version 1.53k). Leaves were then placed in a blast drying oven, dried to constant mass at 65 °C, and weighed to obtain leaf dry mass. Specific leaf weight was calculated as leaf dry mass divided by leaf area.

### 2.3. Leaf Beetle Field Survey

We surveyed the occurrence and abundance of the two leaf beetle species on both willow hosts in July 2025. To quantify host abundance and beetle occurrence, we established five transects (each 35 m × 35 m, covering 1225 m^2^ per transect) within the study site. Occurrence frequency was recorded as presence/absence on each surveyed tree, regardless of abundance. For population density estimation, we randomly selected and observed 11 SW clumps and 6 LW clumps. Population density was recorded as the number of individuals per tree per encounter, regardless of developmental stage.

### 2.4. Dual-Choice Feeding Preference Assay

Leaves used in bioassays were newly expanded, undamaged, and of uniform age to eliminate variation caused by leaf maturity or mechanical damage. Larval preference between SW and LW was tested using dual-choice assays in Petri dishes. The eggs of *Chrysomela vigintipunctata alticola* (CV) are typically pale yellow, clustered, and smooth, while those of *Phratora vitellinae* (PV) are white, arranged in two rows, and enclosed by an egg membrane (see Graphic Abstract). These distinct morphological characteristics allowed us to easily differentiate the eggs and confirm the species of hatching larvae, enabling accurate assignment to the four larval groups: CVS (CV larvae originating from and reared on SW), CVL (CV larvae originating from and reared on LW), PVS (PV larvae originating from and reared on SW), and PVL (PV larvae originating from and reared on LW). For each group, 18 larvae originating from at least five independent egg clutches (≥3 larvae per clutch) were randomly selected in July 2025 for the bioassays. Larvae from each group were individually placed in the center of separate Petri dishes, positioned equidistant from SW and LW leaf discs (Supplementary Figures S1–S4). Choice behavior was recorded at 1 h and 12 h after release. Larvae that selected LW were recorded as choosing “LW”, those that selected SW as choosing “SW”, and individuals that remained at the dish edge without choosing either leaf were defined as “no choice” and excluded from statistical analysis.

### 2.5. Larval Morphology and Instar Duration

To evaluate feeding differentiation and larval performance, larvae from the four groups were reared separately on their respective natal-host leaves under controlled laboratory conditions (room temperature: 14–21 °C; relative humidity: 44–58%; ambient natural daylight under laboratory roof conditions). Specifically, CVL and PVL were reared on LW, while CVS and PVS were reared on SW. Fresh leaves were provided daily to maintain food quality and prevent mold growth. Larvae were inspected four times each day to track molting timing and quantify morphological traits at standardized initial and late instar stages. When individuals from distinct egg clutches reached each instar time point, whole larvae were individually selected and fixed in pre-warmed Carnoy’s solution (60–70 °C water bath) to fully expand and retain intact body morphological features. After fixation, specimens were preserved in 75% ethanol for subsequent microscopic imaging. Whole larvae were photographed under a stereomicroscope with a ruler included as a scale bar. Body length and head capsule width were then measured from the images using ImageJ software (Version 1.53k) at the initial (newly hatched or newly molted) and late (just prior to molting) stages of each larval instar. Additionally, we measured the instar duration of each instar for the four larval types under identical rearing conditions. Instar duration was defined as the period from larval hatching to the first molt, or from one molt to the next molt. Due to destructive sampling for morphological measurements, sample sizes decreased progressively as larvae developed (see Supplementary Tables S1 and S2).

### 2.6. Leaf and Insect Chemical Analysis

We quantified leaf total phenolics, salicylate esters, and total nitrogen, as well as whole-body total nitrogen in adult beetles. At least 50 fresh leaves were randomly collected as described above from SW and 30 from LW. All leaves were oven-dried at 65 °C to a constant weight for chemical analysis. Given that the individual leaf mass of SW is lower than that of LW, we adopted a mixed pooling strategy to ensure sufficient sample mass for each assay. Specifically, five randomly selected SW leaves were pooled as one biological replicate, whereas a single individual LW leaf constituted one replicate. Dried leaf samples were ground into powder, soaked in 60% ethanol, and extracted ultrasonically at 75 °C for 30 min using a KQ3200B ultrasonic cleaner (Kunshan Ultrasonic Instruments Co., Ltd., Kunshan, China) operated at 40 kHz and 150 W. The obtained extracts were subsequently processed for quantification of total phenols and salicylate esters.

The total phenolic content in the plant leaves was determined using the Folin–Ciocalteu method [34] with a commercial kit (Shaanxi Yirongdacheng Biotechnology Co., Ltd., Xixian New Area, Shaanxi, China; instructions available at www.sxyrdc.com). Total salicylate esters were quantified indirectly via acid hydrolysis coupled with FeCl_3_-based colorimetric detection of free salicylic acid. Briefly, the ethanolic extracts were acidified with 2 mol/L hydrochloric acid, heated in a water bath for 10 min, mixed with FeCl_3_ chromogenic reagent, and measured colorimetrically using a UV–visible spectrophotometer (Model UV-1500, Macylab Instruments Co., Ltd., Shanghai, China). This acid hydrolysis step cleaves all salicin-type glycosidic bonds and converts salicin and its derivatives (e.g., salicortin, tremulacin, fragilin) into free salicylic acid; thus, the method cannot distinguish salicin from other salicylic acid conjugates. The resulting measurement quantifies the total pool of salicylate esters, a proxy for the total concentration of herbivore-deterrent salicinoid defensive metabolites in willow leaves. For both total phenolic and salicylate esters assays, 9 biological replicates were prepared per willow species.

Total nitrogen was extracted via concentrated sulfuric acid digestion and quantified using a commercial nitrogen quantification kit from the same manufacturer (Shaanxi Yirongdacheng Biotechnology Co., Ltd.) used for plant samples. Similarly to the phenolic and salicin assays, leaf total nitrogen was measured using 9 biological replicates per willow species. Adult leaf beetles were collected mainly from their respective host plants: CV adults from SW and PV adults from LW. Three dried adults were pooled as one biological replicate, resulting in 5 replicates for each beetle species (CV and PV). Because the sulfuric acid digestion method is applicable to both plant and animal matrices, this nitrogen kit was employed to measure total nitrogen in adult beetles.

### 2.7. Data Analysis

Feeding preference data were analyzed using the binomial exact test to determine whether the proportion of larvae choosing LW differed significantly from the null expectation of 0.5 (random choice).

Independent t-tests were used to evaluate differences in abundance and leaf size between the two willow species, occurrence frequency, population density, and leaf chemical traits between the two willow species or the two beetle species. Instar duration was also compared using independent t-tests.

Morphological traits (log-transformed body length and head capsule width) were analyzed using general linear models (GLMs) at each developmental stage. Fixed factors included larval group (CVS, CVL, PVS, PVL) and developmental stage (a combined variable of instar and instar time point), with the interaction term Group × Stage incorporated to allow host-induced morphological differences to vary across larval developmental stages. Within each identical developmental stage, two pre-planned biologically relevant contrasts (CVL vs. CVS, and PVL vs. PVS) were conducted. All *p*-values from pairwise contrasts were adjusted via the false discovery rate (FDR) method to correct for multiple comparisons across six developmental stages.

All statistical analyses were performed in R 4.5.3 [32], with *p* < 0.05 considered statistically significant.

## 3. Results

### 3.1. Host Abundance and Leaf Size

**Figure 2:**
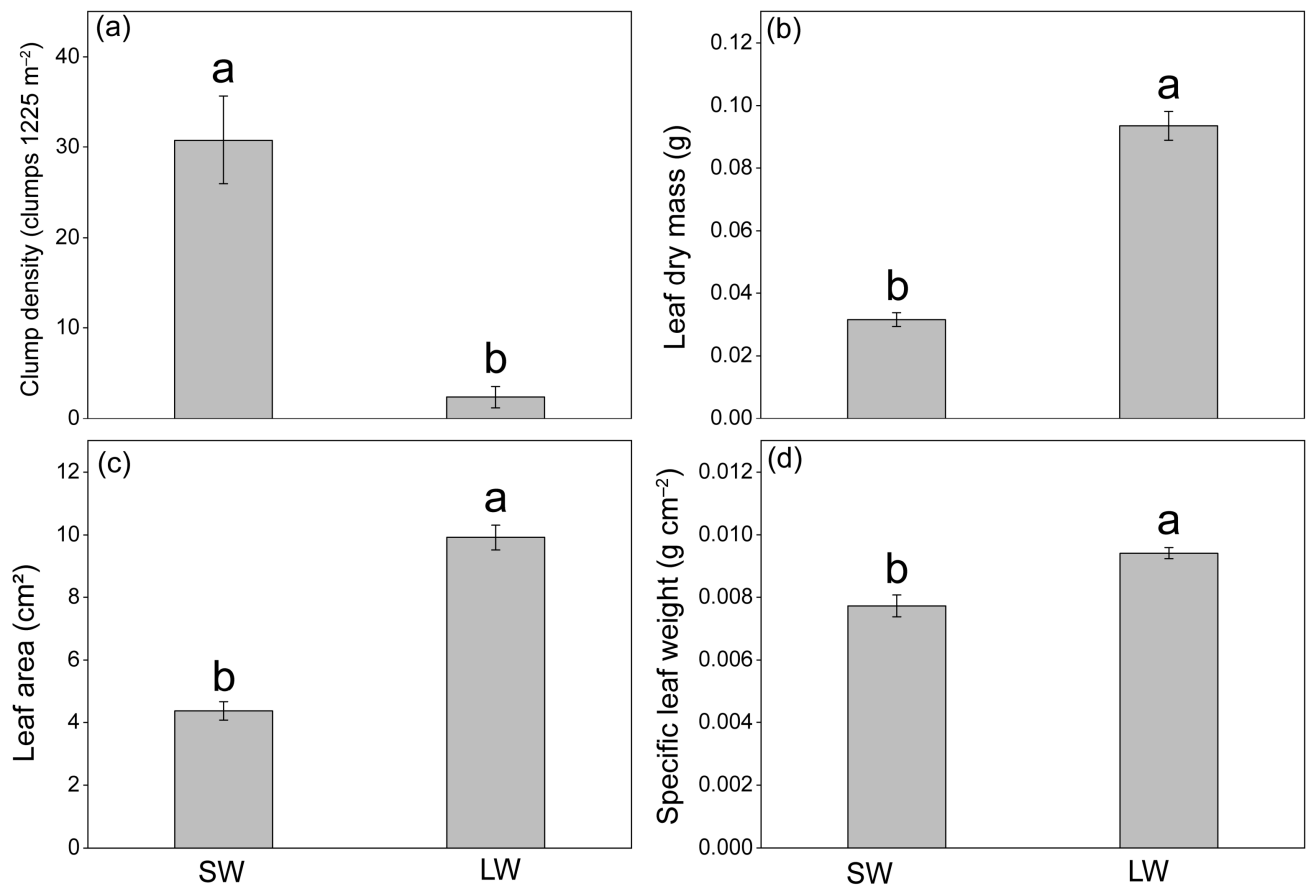
Abundance and leaf size of the two willow species. (**a**) Clump abundance of small-leaf willow (SW) and large-leaf willow (LW) in the field plots (clumps per 1225 m^2^). (**b**) Leaf dry mass (g). (**c**) Leaf area (cm^2^). (**d**) Specific leaf weight (g cm^−2^). Values are presented as mean ± SE. Different lowercase letters indicate significant differences between the two willow species (*p* < 0.05).

Field surveys showed the clump abundance of SW was significantly higher than that of LW (95% CI = [0.726, 1.327], Cohen’s d = 4.64, *p* < 0.001; Figure 2a). Leaf dry mass, leaf area, and specific leaf weight were all significantly lower in SW than in LW (leaf dry mass: 95% CI = [0.112, 0.149], Cohen’s d = 3.261, *p* < 0.001; leaf area: 95% CI = [0.263, 0.369], Cohen’s d = 3.088, *p* < 0.001; specific leaf weight: 95% CI = [0.005, 0.014], Cohen’s d = 1.129, *p* < 0.001; Figure 2b–d).

### 3.2. Field Distribution of Leaf Beetles

**Figure 3:**
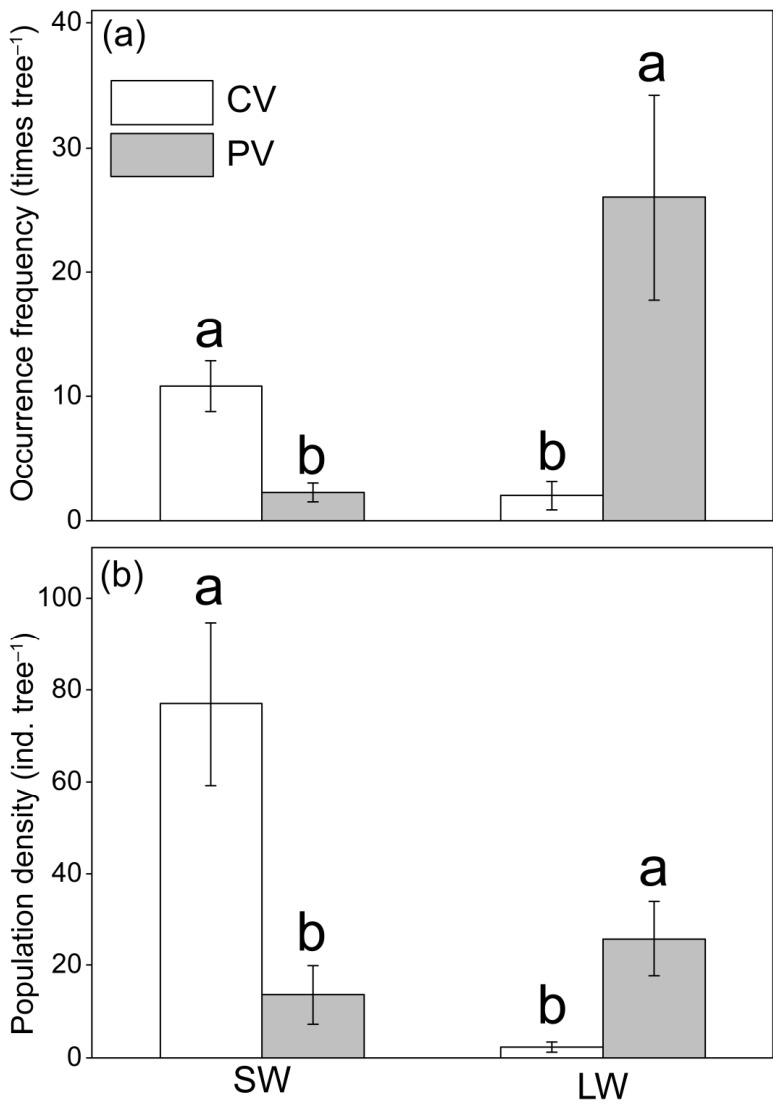
Occurrence frequency (**a**) and population density (**b**) of two leaf beetle species on small-leaf willow (SW) and large-leaf willow (LW). CV, *Chrysomela vigintipunctata alticola*, PV, *Phratora vitellinae*. Different lowercase letters indicate significant pairwise differences within each host plant (*p* < 0.05).

Both occurrence frequency and population density of CV on SW were significantly higher than those of CV on LW (occurrence frequency: 95% CI = [0.339, 0.89], Cohen’s d = 1.997, *p* < 0.001; population density: 95% CI = [0.601, 1.561], Cohen’s d = 2.068, *p* < 0.001; Figure 3). For PV, the occurrence frequency and population density on SW were significantly lower than those on LW (occurrence frequency: 95% CI = [0.582, 1.459], Cohen’s d = 3.04, *p* < 0.001; population density: 95% CI = [0.533, 1.349], Cohen’s d = 2.996, *p* < 0.001; Figure 3).

### 3.3. Larval Feeding Preference

At 1 h, CVS (CV larvae originating from and reared on SW) and CVL (CV larvae originating from and reared on LW) larvae showed no significant preference between SW and LW (*p* > 0.05). In contrast, PVS (PV larvae originating from and reared on SW) and PVL (PV larvae originating from and reared on LW) larvae exhibited a strong, significant preference for LW (*p* = 0.003 and *p* = 0.021; Table 1).

After 12 h, CVS larvae remained non-selective (*p* > 0.05), whereas CVL larvae developed a significant, albeit delayed, preference for LW (*p* = 0.013). Both PVS and PVL larvae maintained a strong preference for LW (both *p* < 0.01; Table 1).

### 3.4. Morphological Responses to Leaf Diet

**Figure 4:**
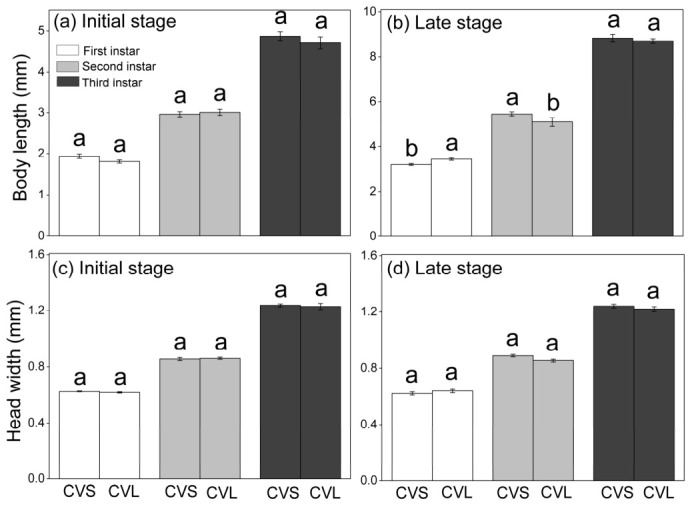
Larval body length and head capsule width between CVL and CVS across instar stages. (**a**) Body length at the initial stage of each instar; (**b**) Body length at the late stage of each instar; (**c**) Head capsule width at the initial stage of each instar; (**d**) Head capsule width at the late stage of each instar. CVS, *Chrysomela vigintipunctata alticola* larvae from SW; CVL, *C. vigintipunctata alticola* larvae from LW. Different lowercase letters indicate significant differences between the two host diet treatments (*p* < 0.05).

Body length did not differ between CVS (CV larvae originating from and reared on SW) and CVL (CV larvae originating from and reared on LW) at the initial stage of the first instar (95% CI = [−0.041, 0.002], Cohen’s d = 0.597, *p* = 0.086; Figure 4a), but was significantly greater in CVL than in CVS at the late stage of the first instar (95% CI = [0, 0.049], Cohen’s d = 1.134, *p* = 0.049; Figure 4b). Overall, no significant difference was detected between CVS and CVL in the second and third instars (initial second instar: 95% CI = [−0.018, 0.029], Cohen’s d = 0.162, *p* = 0.608; initial third instar: 95% CI = [−0.041, 0.017], Cohen’s d = 0.327, *p* = 0.357; late third instar: 95% CI = [−0.028, 0.018], Cohen’s d = 0.157, *p* = 0.629; Figure 4a,b), with the exception that CVS was greater than CVL in the late stage of the second instar (95% CI = [−0.051, −0.003], Cohen’s d = 0.672, *p* = 0.011; Figure 4b).

For head capsule width, no significant difference was detected between CVS and CVL at either the initial or late stages of any instar (initial first instar: 95% CI = [−0.011, 0.007], Cohen’s d = 0.358, *p* = 0.594; late first instar: 95% CI = [−0.004, 0.014], Cohen’s d = 0.352, *p* = 0.26; initial second instar: 95% CI = [−0.008, 0.011], Cohen’s d = 0.128, *p* = 0.741; late second instar: 95% CI = [−0.017, 0.001], Cohen’s d = 0.887, *p* = 0.056; initial third instar: 95% CI = [−0.013, 0.01], Cohen’s d = 0.159, *p* = 0.74; late third instar: 95% CI = [−0.013, 0.005], Cohen’s d = 0.306, *p* = 0.277; Figure 4c,d).

**Figure 5:**
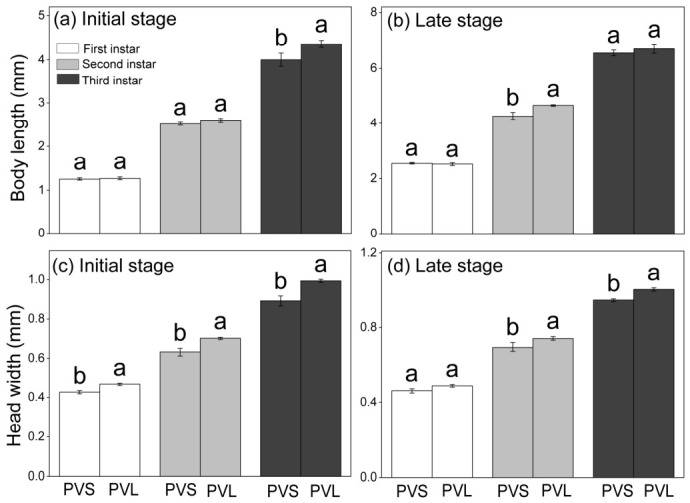
Larval body length and head capsule width between PVS and PVL across instar stages. (**a**) Body length at the initial stage of each instar; (**b**) Body length at the late stage of each instar; (**c**) Head capsule width at the initial stage of each instar; (**d**) Head capsule width at the late stage of each instar. PVS, *Phratora vitellinae* larvae from SW; PVL, *P. vitellinae* larvae from LW. Different lowercase letters indicate significant differences between the two host diet treatments (*p* < 0.05).

No significant difference in body length was observed between PVL (PV larvae originating from and reared on LW) and PVS (PV larvae originating from and reared on SW) at either the initial or late stages of the first instar (initial first instar: 95% CI = [−0.024, 0.028], Cohen’s d = 0.09, *p* = 0.85; late first instar: 95% CI = [−0.029, 0.021], Cohen’s d = 0.171, *p* = 0.695; Figure 5a,b). During the second instar, body length did not differ between groups at the initial stage (95% CI = [−0.016, 0.033], Cohen’s d = 0.365, *p* = 0.608), but PVS was significantly shorter than PVL at the late stage (95% CI = [0.009, 0.057], Cohen’s d = 1.037, *p* = 0.004). In the third instar, PVS remained significantly shorter than PVL at the initial stage (95% CI = [0.01, 0.057], Cohen’s d = 0.725, *p* = 0.003), with no intergroup difference observed at the late stage (95% CI = [−0.014, 0.029], Cohen’s d = 0.209, *p* = 0.629; Figure 5a,b).

For head capsule width, PVL was significantly wider than PVS at the initial stage of the first instar (95% CI = [0.003, 0.023], Cohen’s d = 1.527, *p* = 0.01; Figure 5c), but did not differ from PVS at the late stage of first instar (95% CI = [−0.011, 0.019], Cohen’s d = 0.836, *p* = 0.094; Figure 5d). In contrast, PVL larvae exhibited consistently wider head capsules than PVS larvae at both the initial and late stage of the second and third instars (initial second instar: 95% CI = [0.009, 0.029], Cohen’s d = 1.117, *p* < 0.001; late second instar: 95% CI = [0.003, 0.022], Cohen’s d = 0.681, *p* = 0.01; initial third instar: 95% CI = [0.006, 0.025], Cohen’s d = 0.698, *p* < 0.001; late third instar: 95% CI = [0.005, 0.022], Cohen’s d = 1.606, *p* < 0.001; Figure 5c,d).

### 3.5. Larval Instar Duration

**Figure 6:**
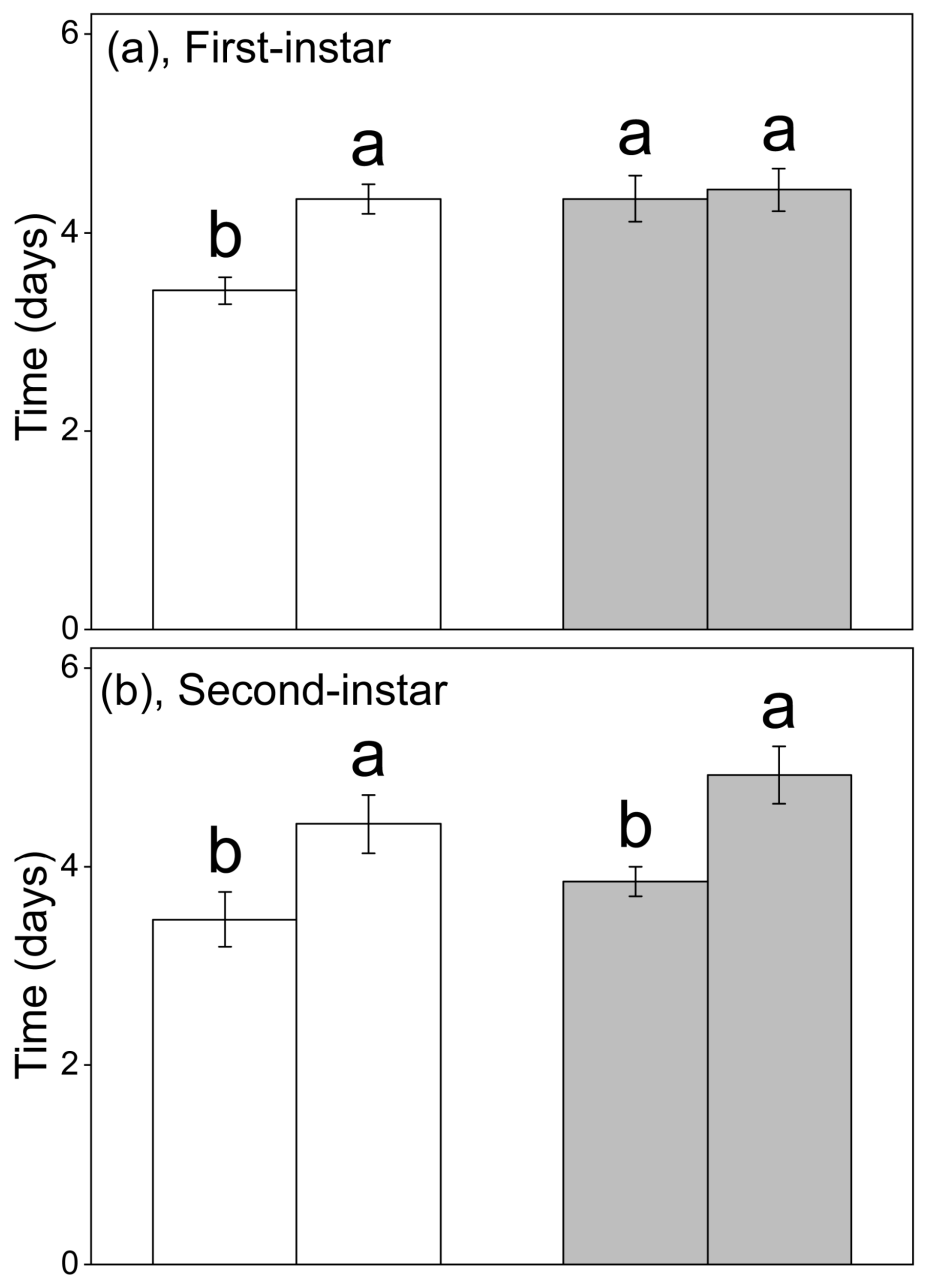

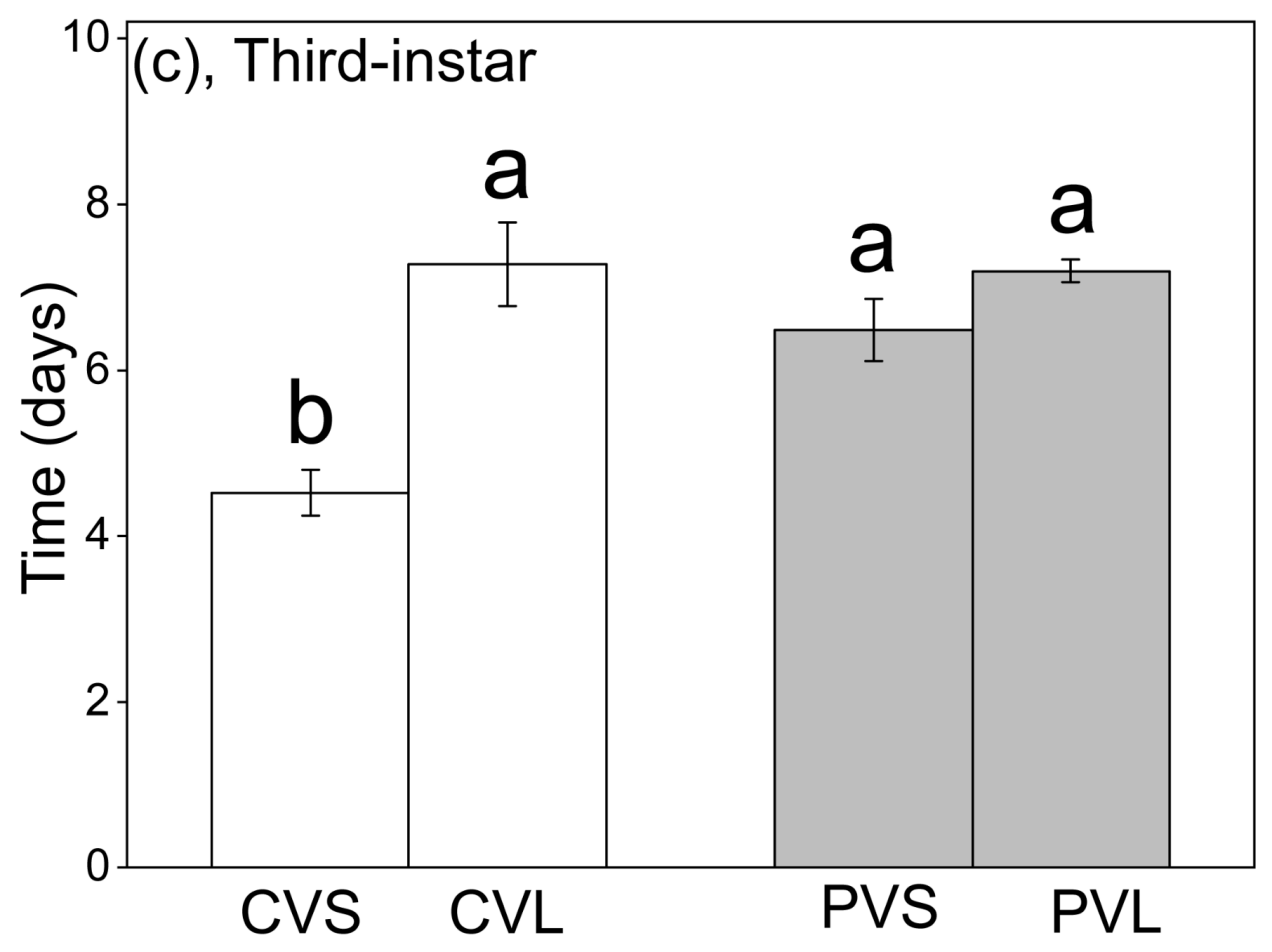
Larval instar duration (days) of four larval groups fed on their natal-host willow foliage across three instars. (**a**) Instar duration of the first instar; (**b**) Instar duration of the second instar; (**c**) Instar duration of the third instar. CVS, *Chrysomela vigintipunctata alticola* larvae from SW; CVL, *C. vigintipunctata alticola* larvae from LW; PVS, *Phratora vitellinae* larvae from SW; PVL, *P. vitellinae* larvae from LW. Different lowercase letters indicate significant pairwise differences between CVS and CVL, and between PVS and PVL within each instar (*p* < 0.05).

The duration of all three instars was significantly longer for CVL (CV larvae originating from and reared on LW) than for CVS (CV larvae originating from and reared on SW) (first-instar: 95% CI = [0.045, 0.121], Cohen’s d = 1.969, *p* < 0.001; second-instar: 95% CI = [0.033, 0.147], Cohen’s d = 1.515, *p* < 0.001; third-instar: 95% CI = [0.098, 0.257], Cohen’s d = 2.717, *p* < 0.001; Figure 6). The duration of the first and third instars did not differ between PVS (PV larvae originating from and reared on SW) and PVL (PV larvae originating from and reared on LW) (first-instar: 95% CI = [0.047, 0.062], Cohen’s d = 0.122, *p* = 0.79; third-instar: 95% CI = [0.014, 0.097], Cohen’s d = 1.072, *p* = 0.12; Figure 6a,c), whereas the duration of the second instar was significantly longer for PVL than for PVS (second-instar: 95% CI = [0.028, 0.14], Cohen’s d = 1.513, *p* = 0.006; Figure 6b).

### 3.6. Leaf Chemical Traits and Adult Nitrogen Content

**Figure 7:**
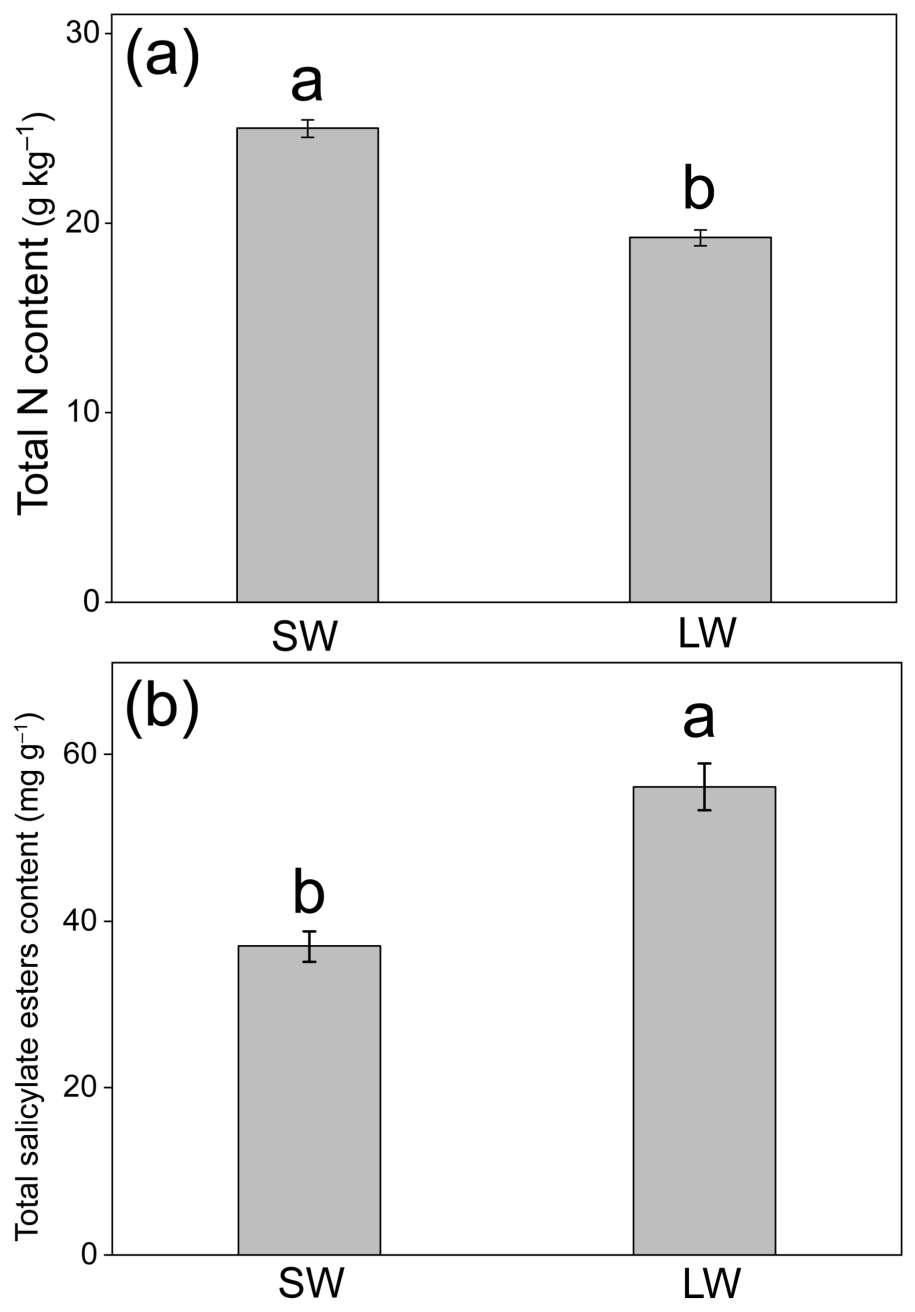

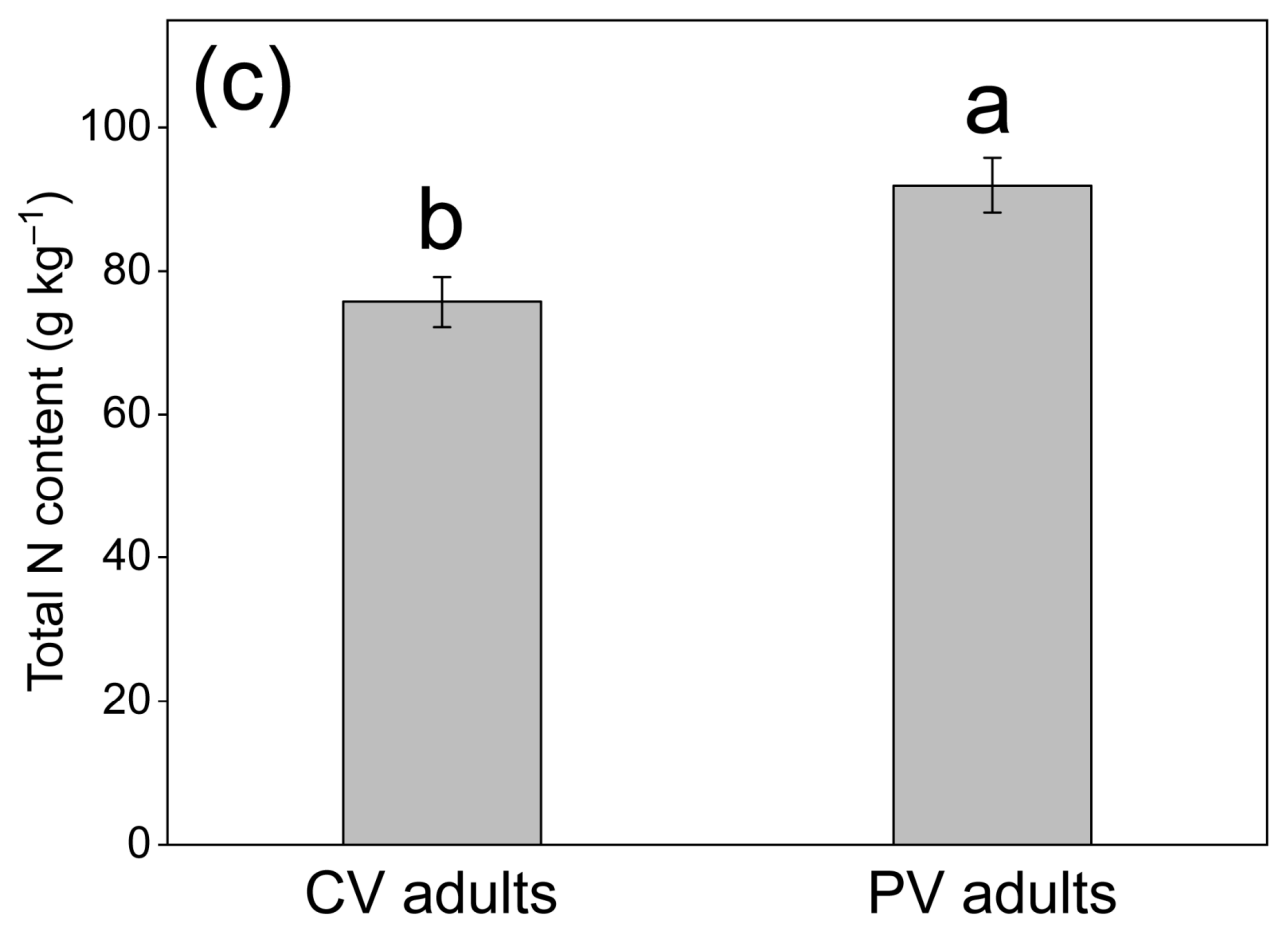
Leaf total nitrogen content (**a**), leaf total salicylate ester content (**b**), and whole-body total nitrogen content of CV and PV adult beetles (**c**). Different lowercase letters above error bars indicate statistically significant differences between the two willow species (**a**,**b**) or between the two beetle species (**c**) (*p* < 0.05).

Total phenolic content did not differ significantly between SW and LW (95% CI = [0.021, 0.044], Cohen’s d = 0.358, *p* = 0.458; Supplementary: Figure S5). In contrast, total salicylate esters content was significantly higher in LW than in SW (95% CI = [0.082, 0.082], Cohen’s d = 1.823, *p* = 0.001; Figure 7a). Total leaf nitrogen content was significantly higher in SW than in LW (95% CI = [0.081, 0.13], Cohen’s d = 4.262, *p* < 0.001; Figure 7b), while adult total nitrogen content was significantly lower in CV than in PV (95% CI = [0.017, 0.122], Cohen’s d = 1.712, *p* = 0.014; Figure 7c).

## 4. Discussion

Our study provides empirical evidence for fine-scale feeding preference differentiation and niche partitioning between two sympatric willow leaf beetles, *Chrysomela vigintipunctata alticola* and *Phratora vitellinae*, in an alpine meadow ecosystem. This divergence—characterized by PV’s strong association with LW (large-leaved willow) and CV’s reliance on SW (small-leaved willow)—is associated with interspecific differences in leaf functional traits (leaf size, total nitrogen, and defensive secondary metabolite concentrations). By examining larval innate feeding preference and developmental performance, we clarified the mechanisms underlying adult host selection in the field: PV larvae exhibit a strong intrinsic feeding response for LW, which also maximizes their morphological growth, reflecting coupling between host preference and larval fitness, whereas CV larvae develop significantly faster on SW but lack a strong intrinsic feeding response, suggesting that adult oviposition on SW is a life-history compromise driven by temporal constraints and host availability. Divergent responses in larval morphology, instar duration and resource use further facilitate local resource partitioning and co-occurrence of the two species. Overall, our experimental results partially supported the preference-performance hypothesis: PV exhibited close coupling between intrinsic feeding response and larval fitness, whereas CV displayed a partial decoupling, rejecting our predictions that CV would show a consistent intrinsic feeding response and uniform growth advantages on SW. Our findings align with modern coexistence theory and recent community ecology frameworks emphasizing that coexisting herbivores partition resources along multiple niche axes, including food quality, defensive traits, and spatial availability, rather than merely using different hosts [5,10,35], consistent with evidence that host plant traits can drive resource partitioning even at the intraspecific level in polyphagous insects [36].

PV larvae exhibited a strong and consistent preference for LW at both 1 h and 12 h, which was highly consistent with their field distribution pattern. This preference was closely linked with enhanced morphological performance, as evidenced by greater body length at specific developmental time points and consistently wider head capsules across all instars. These results support the preference-performance hypothesis [27,28], which posits that females oviposit on hosts that maximize offspring fitness. Such strong coupling between feeding preference and larval performance and fitness is particularly common in chemically specialized herbivores, where host choice directly determines survival and development [28], and is widely documented in leaf beetles associated with willow hosts [1,37]. This indicates that PV adults actively choose LW to match their offspring’s intrinsic feeding response, thereby optimizing fitness through specific host adaptation.

In contrast to PV, our predictions that CV larvae would exhibit a strong intrinsic preference for SW and achieve uniform growth advantages on this host were not supported. CVS showed no intrinsic feeding response for either leaf type, while CVL developed a delayed preference for LW after 12 h, suggesting behavioral plasticity influenced by their natal host environment. This weak delayed preference likely reflects a transient behavioral plasticity influenced by natal host chemical exposure, rather than a stable innate preference, as it was absent in CVS larvae and did not translate into consistent growth advantages across all instars. Nevertheless, morphological data indicated that CV larvae exhibited inconsistent and transient differences on either host (e.g., greater body length on LW at the late first instar, but greater body length on SW at the late second instar), yet adult CV primarily oviposit on SW in the field. This discrepancy suggests that larval growth traits are not the decisive factor driving adult host selection, highlighting a partial decoupling between larval performance and adult oviposition choice—a contrasting pattern increasingly documented in herbivorous insects experiencing strong ecological constraints [28,38], which often arises because adults and larvae experience distinct selective pressures, including predation, microclimate, and resource availability. This decoupling indicates that host selection in alpine insects represents a balanced outcome of multiple selective pressures. Specifically, although CVL larvae may exhibit a weak delayed preference for the initially growth-promoting LW under controlled conditions, adult CV in the wild face strong selection to oviposit on SW, where rapid development ensures offspring survival before winter.

We propose that life-history trade-offs between developmental rate and body size underlie these contrasting selection patterns. Although LW may offer transient early morphological benefits, it does not confer a consistent growth advantage for CV; our instar duration data revealed a contrasting pattern: CV larvae reared on SW developed significantly faster across all three instars. On the Qinghai–Tibet Plateau, the growing season is short (approximately July to August), and the approaching winter frost imposes strong time constraints on insect development [23]. For CV, prioritizing developmental rate to ensure timely pupation before winter likely confers a higher selective advantage than maximizing final body size. This interpretation is consistent with life-history theory predicting strong selection for rapid development in seasonal, high-altitude habitats [39,40], where incomplete development before frost causes high mortality [40]. Such a developmental rate vs. body size trade-off is a common life-history strategy for herbivores inhabiting seasonally constrained habitats, reflecting ecological trade-offs shaped by limited growing seasons [41]. Conversely, PV experiences a significant developmental delay only during the second instar on LW; PV larvae can tolerate this localized time cost because feeding on LW confers consistent gains in head capsule width across all larval stages. Collectively, these divergent phenotypic patterns possibly reflect ecological trade-offs shaped by the same harsh environment through divergent life-history trait combinations.

The divergent developmental rates observed between CV fed on SW and LW can be largely attributed to differences in leaf nitrogen concentration. Leaf total nitrogen was significantly higher in SW than in LW, indicating that SW represents a more nutritious host. This higher nitrogen availability likely contributes to the faster developmental rate of CV on SW, consistent with the well-established expectation that increased host-plant nitrogen alleviates nutritional constraints and thereby accelerates larval development in herbivorous insects [42,43,44]. For PV, despite lower nitrogen in LW, larvae still achieved better morphological performance, suggesting PV possesses specialized physiological adaptations to efficiently utilize nutrients from LW and maintain growth without severe developmental penalties. Moreover, PV adults exhibit a higher observed nitrogen content than CV adults, suggesting potential physiological adaptations for nitrogen assimilation and retention, enabling it to accumulate substantial nitrogen reserves despite feeding on a low-nitrogen host. In contrast, despite feeding on nitrogen-rich SW, CV adults exhibit lower nitrogen content, likely because they prioritize converting acquired nitrogen into reproductive output and metabolic turnover rather than long-term retention.

Beyond nutritional constraints, plant secondary metabolites—especially phenolics and salicylates—are well known to mediate host recognition and feeding choice in willow-feeding leaf beetles [45,46]. Phenolic compounds can act as both feeding deterrents and sequestrable defenses, shaping specialist herbivore host preference [1]. Our chemical assays confirmed that LW contained significantly higher total salicylate esters content than SW, whereas total phenolic content did not differ between the two hosts. (Supplementary Figure S5). This contrast indicates that interspecific defensive divergence between the two willow species is driven primarily by varying salicinoid concentrations rather than total phenolic levels, further highlighting the central role of salicylate esters in mediating host resource partitioning for these two leaf beetles. While our salicylate measurements represent the total pool of salicinoids, the specific composition and bioavailability of individual salicylate esters may further modulate PV’s feeding preference and sequestration efficiency. As a specialist herbivore strongly dependent on salicylate-rich willows, PV relies on salicin-rich resources to sequester defensive chemicals [18,47], while *Chrysomela* species show weak dependence on host salicylate esters [47]. Given the potential role of gut microbiota in salicin detoxification and sequestration efficiency [48], interspecific divergence in microbial communities may further underpin the distinct host-use strategies observed in PV and CV. The strong preference of PV for LW is therefore most likely driven by a need to acquire these defensive compounds. These divergent host-use strategies reflect distinct evolutionary specializations rather than a simple suppression of insect defense by high plant nitrogen [49,50]. Unable to sequester salicin, CV capitalizes on the high-nitrogen SW as a nutritional reservoir to prioritize rapid development. This leads to a fundamental difference in their host-use strategies: PV possesses specialized physiological pathways to sequester salicin [47], and its preference for high-salicylate LW demonstrates that the fitness benefits of chemical defense outweigh the growth costs imposed by lower nutritional quality. In contrast, CV larvae lack an intrinsic feeding response for SW. We attribute this lack of innate preference to the nature of the cues involved: salicin acts as a fixed chemical signal that drives stable host recognition in PV, whereas leaf nitrogen content is highly dynamic and changes with plant phenology and environmental conditions [12,44]. Therefore, nitrogen may not be a stable enough cue to drive a strong innate larval preference in CV. Recent work has revealed that specialist leaf beetles often evolve enhanced detoxification or sequestration pathways for utilizing salicylate-rich hosts, further reinforcing host specialization [37,51,52]. Such host-specific associations are thought to be mediated by targeted recognition of specific plant metabolites.

Divergent pupation strategies may further explain the species’ contrasting defensive chemical requirements. CV pupae develop externally on plant tissues with a relatively sclerotized cuticle [19], with typically low parasitism rates by chalcid wasps [53]. In contrast, PV pupates mainly in soil, with a soft, unsclerotized pupal case [20]. Since this species relies entirely on sequestered host-plant chemicals for defense and lacks strong physical pupal protection [37], it is far more vulnerable to soil predators and pathogens. We infer that this elevated soil-associated predation risk selects for PV’s strong preference for high-salicylate LW, even when this host contains lower leaf nitrogen and brings minor developmental costs.

It is important to acknowledge that our fitness assessment focused on larval performance rather than full life-cycle parameters as established in previous studies on *Chrysomela* [54], which included survival rates from hatching to adult emergence and newly emerged adult weight. Because this study required destructive sampling at each instar for detailed morphological measurements, the number of individuals decreased progressively, limiting the number of individuals reaching adulthood for statistical analysis. Nevertheless, the larval stages represent the critical period for host association and niche partitioning, and the parameters measured in this study provide sufficient evidence to address our specific hypotheses regarding life-history trade-offs. Nevertheless, future studies incorporating full life-cycle parameters would provide a more complete understanding of the fitness trade-offs in this system.

While our single-generation natal-host rearing trials approximate field larval feeding conditions, it should be noted that this experimental setup cannot fully disentangle maternal carry-over effects and transgenerational plasticity from direct dietary impacts on larval behavioral and performance traits. Although our results show that CVL larvae developed a delayed preference for LW, this behavioral pattern may not necessarily reflect a purely intrinsic feeding response. Instead, it could potentially originate from maternal chemical or nutritional signals deposited in eggs by LW-reared maternal adults. Similarly, maternal provisioning can also shape larval developmental performance independent of larval feeding experience. For example, CV females on nitrogen-rich SW may invest more resources into eggs, providing their offspring with larger initial reserves that accelerate development irrespective of the larvae’s own diet. Future studies employing a full factorial cross-rearing design—rearing larvae originating from both hosts on both SW and LW—are required to isolate maternal-origin effects from direct larval dietary induction [55,56]. Such reciprocal experiments are needed to determine whether the observed life-history trade-offs are driven primarily by the direct nutritional and chemical constraints of the current larval diet, or indirectly via transgenerational plasticity shaped by the maternal environment.

## 5. Conclusions

In summary, host plant trait variation (leaf size, nitrogen, and salicylate esters) creates distinct resource gradients that allow sympatric herbivores to co-occur locally. By using larval preference to infer adult oviposition strategies, we revealed that these two beetles optimize distinct fitness priorities: PV adults actively select LW to match offspring preference and maximize chemical defense, whereas CV adults exploit the abundant SW, prioritizing rapid development over inconsistent morphological growth under extreme temporal constraints. These findings demonstrate that niche partitioning in alpine herbivores emerges from the interplay of behavioral preference and physiological trade-offs. Future research integrating multi-omics (e.g., transcriptomics and metagenomics) could elucidate the genetic and microbial basis of salicin sequestration and developmental plasticity in these beetles. Furthermore, long-term monitoring and manipulative experiments assessing how shifting growing seasons and climate warming alter these host-use dynamics will be crucial for predicting the stability and resilience of alpine insect communities.

## Figures and Tables

**Table 1:** Larval preference of four beetle groups (CVS, CVL, PVS, PVL) in dual-choice bioassays. Preference was determined using binomial exact tests against a null hypothesis of 0.5 (random choice). CVS, *Chrysomela vigintipunctata alticola* larvae from SW; CVL, *C. vigintipunctata alticola* larvae from LW; PVS, *Phratora vitellinae* larvae from SW; PVL, *P. vitellinae* larvae from LW. * *p* < 0.05, ** *p* < 0.01, ns, not significant. Individuals making no choice were excluded.

Time Point	Group	LW	SW	*p*
1 h	CVS	9	4	0.267 ns
1 h	CVL	12	4	0.077 ns
1 h	PVS	12	1	0.003 **
1 h	PVL	11	2	0.021 *
12 h	CVS	10	7	0.629 ns
12 h	CVL	14	3	0.013 *
12 h	PVS	14	2	0.004 **
12 h	PVL	11	1	0.006 **

## Data Availability

The original contributions presented in this study are included in the article together with the Supplementary Materials. Further inquiries can be directed to the corresponding author.

## Supplementary Materials

The following supporting information can be downloaded at: https://www.mdpi.com/article/10.3390/ani16152395/s1, Table S1. Sample sizes (*n*) for morphological measurements (body length and head capsule width) at initial and late stages of the 1st, 2nd, and 3rd instars for each larval group. CVL = CV larvae originating from and reared on LW; CVS = CV larvae originating from and reared on SW; PVL = PV larvae originating from and reared on LW; PVS = PV larvae originating from and reared on SW. Table S2. Sample sizes (*n*) for instar duration measurements. Figure S1. Representative snapshots of leaf size choice assays for CVS larvae, captured from continuous video recordings. Two independent replicates are shown for each time point: (a,b) at 1 h post-release, and (c,d) at 12 h post-release. Each dish contained one large and one small leaf disc, with red arrows indicating the larva’s location. Figure S2. Representative snapshots of leaf size choice assays for CVL larvae, captured from continuous video recordings. Two independent replicates are shown for each time point: (a,b) at 1 h post-release, and (c,d) at 12 h post-release. Each dish contained one large and one small leaf disc, with red arrows indicating the larva’s location. Figure S3. Representative snapshots of leaf size choice assays for PVS larvae, captured from continuous video recordings. Two independent replicates are shown for each time point: (a,b) at 1 h post-release, and (c,d) at 12 h post-release. Each dish contained one large and one small leaf disc, with red arrows indicating the larva’s location. Figure S4. Representative snapshots of leaf size choice assays for PVL larvae, captured from continuous video recordings. Two independent replicates are shown for each time point: (a,b) at 1 h post-release, and (c,d) at 12 h post-release. Each dish contained one large and one small leaf disc, with red arrows indicating the larva’s location. Dishes without an arrow in panel (c) indicate that the larva had escaped. Figure S5. Total phenolic content between SW and LW. Different lowercase letters above bars denote statistically significant differences between the two willow species (*p* < 0.05).

## Abbreviations

The following abbreviations are used in this manuscript:

CV	*Chrysomela vigintipunctata alticola*
PV	*Phratora vitellinae*
SW	*Salix atopantha* (small-leaf willow)
LW	*Salix wangiana* var. *tibetica* (large-leaf willow)
CVS	CV larvae originating from and reared on SW
CVL	CV larvae originating from and reared on LW
PVS	PV larvae originating from and reared on SW
PVL	PV larvae originating from and reared on LW

## References

[1] Tahvanainen J., Julkunen-Tiitto R., Kettunen J. (1985). Phenolic glycosides govern the food selection pattern of willow feeding leaf beetles. Oecologia.

[2] Schoonhoven L.M., Van Loon J.J., Dicke M. (2006). Insect-Plant Biology.

[3] Jaenike J. (1990). Host specialization in phytophagous insects. Annu. Rev. Ecol. Syst..

[4] Jones L.C., Rafter M.A., Walter G.H. (2022). Host interaction mechanisms in herbivorous insects—Life cycles, host specialization and speciation. Biol. J. Linn. Soc..

[5] Chesson P. (2000). Mechanisms of maintenance of species diversity. Annu. Rev. Ecol. Syst..

[6] Haddad N.M., Crutsinger G.M., Gross K., Haarstad J., Tilman D. (2011). Plant diversity and the stability of foodwebs. Ecol. Lett..

[7] Potter A.B., Hutchinson M.C., Pansu J., Wursten B., Long R.A., Levine J.M., Pringle R.M. (2022). Mechanisms of dietary resource partitioning in large-herbivore assemblages: A plant-trait-based approach. J. Ecol..

[8] Zhang R., Shi J., Zhang J., Wang D. (2026). Spatial partitioning among three sympatric ungulates in the tibetan plateau: Identifying critical variables. Integr. Zool..

[9] Gokhman V.E. (2025). Patterns and mechanisms of niche partitioning between related parasitoids (Hymenoptera) sharing the same host species. Insects.

[10] Wang D., Cao Z., Liu Y., Li R., Wu R., Wu W., Liu W., Hu X., Xu Y. (2025). DNA metabarcoding illuminates seasonal dietary pattern and niche partitioning by three sympatric herbivores. Ecol. Evol..

[11] Carmona D., Lajeunesse M.J., Johnson M.T.J. (2011). Plant traits that predict resistance to herbivores. Funct. Ecol..

[12] Mezzomo P., Leong J.V., Pokorny V., Jorge L.R., Volfova T., Kozel P., Vodrazka P., Seifert C.L., Aurová K., Moos M. (2025). Effects of pronounced seasonal turnover and intraspecific variation in leaf traits on specialization of insect herbivores associated with six Salicaceae hosts. Oecologia.

[13] Després L., David J.-P., Gallet C. (2007). The evolutionary ecology of insect resistance to plant chemicals. Trends Ecol. Evol..

[14] Peng X., Reichelt M., Baños-Quintana A.P., Rothe B., Feistel F., Kaltenpoth M., Gershenzon J., Unsicker S.B. (2025). Leaf beetles employ tryptophan to detoxify the chemical defenses of poplar trees. Proc. Natl. Acad. Sci. USA.

[15] Runte K., Ziaja D., Müller C. (2025). Diet switching from leaves to flowers—Is this beneficial for the mustard leaf beetle?. J. Chem. Ecol..

[16] Nomura N., Kasai A. (2025). Variation in leaf utilization sites among three *Calystegia* (Solanales: Convolvulaceae)-feeding leaf beetle species (Coleoptera: Chrysomelidae) partly explains differences in competitiveness: A case study of spatial analysis. J. Insect Sci..

[17] Ge S., Cui J., Yang X. (2004). Comparative morphology of two subspecies of *Chrysomela vigintipunctata* (Scopoli) (Coleoptera, Chrysomelidae, Chrysomelinae). Acta Zootaxon. Sin..

[18] Rank N.E., Köpf A., Julkunen-Tiitto R., Tahvanainen J. (1998). Host preference and larval performance of the salicylate-using leaf beetle *Phratora vitellinae*. Ecology.

[19] Kou L., You S., Liu M., Li Y., Liu P., Wang Z., Liu D., Cheng F. (2025). Morphology of *Chrysomela vigintipunctata alticola* Wang (Coleoptera: Chrysomelidae) with references on the immature stage of larvae in an alpine meadow. Zoomorphology.

[20] Urban J. (2006). Occurrence, development and economic importance of *Phratora* (=*Phyllodecta*) *vitellinae* (L.) (Coleoptera, Chrysomelidae). J. For. Sci..

[21] Wang Y.F., Liang E.Y., Lu X.M., Camarero J.J., Babst F., Shen M.G., Peñuelas J. (2021). Warming-induced shrubline advance stalled by moisture limitation on the Tibetan Plateau. Ecography.

[22] Jiang C., Yue L., Jin D., Zou R., Wang Y., Li B., Du X., Huang X., Sun Y., Wang R. (2026). Ancestral and local adaptation contribute to dispersal out of the Qinghai–Tibet Plateau in a bumblebee. Proc. Natl. Acad. Sci. USA.

[23] Cao R., Chen H., Zheng K., Nong D., Jiang M., Zhang Z., Wu X., Xie P. (2025). Effects of climate warming on the performance of *Gynaephora alpherakii* (Lepidoptera: Lymantriidae) larvae in a tibetan alpine meadow. Ecol. Evol..

[24] Culler L.E., Ayres M.P., Virginia R.A. (2015). In a warmer Arctic, mosquitoes avoid increased mortality from predators by growing faster. Proc. R. Soc. B.

[25] Smith J.M., Telemeco R.S., Briones Ortiz B.A., Nufio C.R., Buckley L.B. (2021). High-elevation populations of montane grasshoppers exhibit greater developmental plasticity in response to seasonal cues. Front. Physiol..

[26] Maracahipes L., Bergamini L.L., Sobral F.L., Almeida-Neto M., Cianciaruso M.V., de Araújo W.S. (2025). Leaf traits mediate galling insect frequency on woody plants in a Neotropical savanna. Flora.

[27] Jaenike J. (1978). On optimal oviposition behavior in phytophagous insects. Theor. Popul. Biol..

[28] Gripenberg S., Mayhew P., Parnell M., Roslin T. (2010). A meta-analysis of preference-performance relationships in phytophagous insects. Ecol. Lett..

[29] Cunningham J.P., West S.A., Zalucki M.P. (2001). Host selection in phytophagous insects: A new explanation for learning in adults. Oikos.

[30] Xi X., Zhou W., Li Z., Hu L., Dong Y., Niklas K.J., Sun S. (2021). Rare plant species are at a disadvantage when both herbivory and pollination interactions are considered in an alpine meadow. J. Anim. Ecol..

[31] Kou L., Dong Y., Sun S. (2022). Insect overwintering stages in an alpine meadow in relation to their phylogeny and soil depth. Ann. Zool. Fenn..

[32] R Core Team (2026). R: A Language and Environment for Statistical Computing.

[33] Kou L., Yang N., Yan H., Niklas K.J., Sun S. (2024). Insect root feeders incur negative density-dependent damage across plant species in an alpine meadow. Ecology.

[34] Lamuela-Raventós R.M., Apak R., Capanoglu E., Shahidi F. (2018). Folin–Ciocalteu method for the measurement of total phenolic content and antioxidant capacity. Measurement of Antioxidant Activity & Capacity.

[35] Valladares F., Bastias C.C., Godoy O., Granda E., Escudero A. (2015). Species coexistence in a changing world. Front. Plant Sci..

[36] Cheng Z., Liu Q., Huang X. (2024). Partial correspondence between host plant-related differentiation and symbiotic bacterial community in a polyphagous insect. Animals.

[37] Zvereva E.L., Zverev V., Kruglova O.Y., Kozlov M.V. (2017). Strategies of chemical anti-predator defences in leaf beetles: Is sequestration of plant toxins less costly than de novo synthesis?. Oecologia.

[38] Friberg M., Posledovich D., Wiklund C. (2015). Decoupling of female host plant preference and offspring performance in relative specialist and generalist butterflies. Oecologia.

[39] Eck D.J., Shaw R.G., Geyer C.J., Kingsolver J.G. (2015). An integrated analysis of phenotypic selection on insect body size and development time. Evolution.

[40] Nagy L., Grabherr G. (2009). The Biology of Alpine Habitats.

[41] Nylin S., Gotthard K. (1998). Plasticity in life-history traits. Annu. Rev. Entomol..

[42] Gao Y., Li H., Lu Y. (2025). Bottom-up effects of nitrogen fertilizer on cotton growth and population expansion of *Aphis gossypii* (Hemiptera: Aphididae). J. Econ. Entomol..

[43] Gu H., Wang H., Shangguan Z., Shi H., Zhu J., He J. (2022). Effects of nitrogen addition on the population density of *Gynaephora menyuanensis* in Tibetan alpine grassland. Acta Ecol. Sin..

[44] Mattson W.J. (1980). Herbivory in relation to plant nitrogen content. Annu. Rev. Ecol. Syst..

[45] Barbehenn R.V., Peter Constabel C. (2011). Tannins in plant–herbivore interactions. Phytochemistry.

[46] Rank N.E. (1992). Host plant preference based on salicylate chemistry in a willow leaf beetle (*Chrysomela aeneicollis*). Oecologia.

[47] Kirsch R., Vogel H., Muck A., Vilcinskas A., Pasteels J.M., Boland W. (2011). To be or not to be convergent in salicin-based defence in chrysomeline leaf beetle larvae: Evidence from *Phratora vitellinae* salicyl alcohol oxidase. Proc. R. Soc. B.

[48] Li X., Zhang Y., Chen X., Zhang Y., Khan O., Xu L. (2026). Gut microbiota variation drives differential performance in leaf beetles across host plants. Microbiome.

[49] Bryant J.P., Chapin F.S., Klein D.R. (1983). Carbon/nutrient balance of boreal plants in relation to vertebrate herbivory. Oikos.

[50] Rowell-Rahier M. (1984). The presence or absence of phenolglycosides in *Salix* (Salicaceae) leaves and the level of dietary specialisation of some of their herbivorous insects. Oecologia.

[51] Ma M., Luo J., Chen X., Li C., Li S., Sun J., Xu L. (2024). Gut bacteria facilitate leaf beetles in adapting to dietary specialization by enhancing larval fitness. npj Biofilms Microbiomes.

[52] Dyer L.A., Singer M.S., Lill J.T., Stireman J.O., Gentry G.L., Marquis R.J., Ricklefs R.E., Greeney H.F., Wagner D.L., Morais H.C. (2007). Host specificity of Lepidoptera in tropical and temperate forests. Nature.

[53] Lays P. (2002). Note on mass occurrences of *Chrysomela vigintipunctata* (Scopoli, 1763) (Coleoptera Chrysomelidae Chrysomelinae) in Belgium. Bull. Soc. R. Belge Entomol..

[54] Zvereva E.L., Kozlov M.V., Hilker M. (2010). Evolutionary variations on a theme: Host plant specialization in five geographical populations of the leaf beetle *Chrysomela lapponica*. Popul. Ecol..

[55] Mousseau T.A., Fox C.W. (1998). The adaptive significance of maternal effects. Trends Ecol. Evol..

[56] Marshall D.J., Uller T. (2007). When Is a Maternal Effect Adaptive?. Oikos.

