# Supplementary material for: Feeding Preference Differentiation in Sympatric Willow Leaf Beetles Driven by Host Leaf Trait Divergence
Source: Animals (Basel). 2026 Aug 3;16(15):2395. doi: 10.3390/ani16152395 (PMC13464714; doi:10.3390/ani16152395)
Supplement: Supplementary file 1 [file animals-16-02395-s001.zip › animals-4421833-supplementary.pdf]

Table S1. Sample sizes (*n*) for morphological measurements (body length and head capsule width) at initial and late stages of the 1st, 2nd, and 3rd instars for each larval group. CVL = CV larvae originating from and reared on LW; CVS = CV larvae originating from and reared on SW; PVL = PV larvae originating from and reared on LW; PVS = PV larvae originating from and reared on SW.

Group	1st instar Initial	1st instar Late	2nd instar Initial	2nd instar Late	3rd instar Initial	3rd instar Late
CVL	24	17	18	17	9	21
CVS	23	21	21	22	23	22
PVL	17	24	20	23	21	21
PVS	16	14	17	16	19	29

Table S2. Sample sizes (*n*) for instar duration measurements.

Group	1st instar (*n*)	2nd instar (*n*)	3rd instar (*n*)
CVL	9	8	5
CVS	13	12	9
PVL	11	10	7
PVS	9	8	6

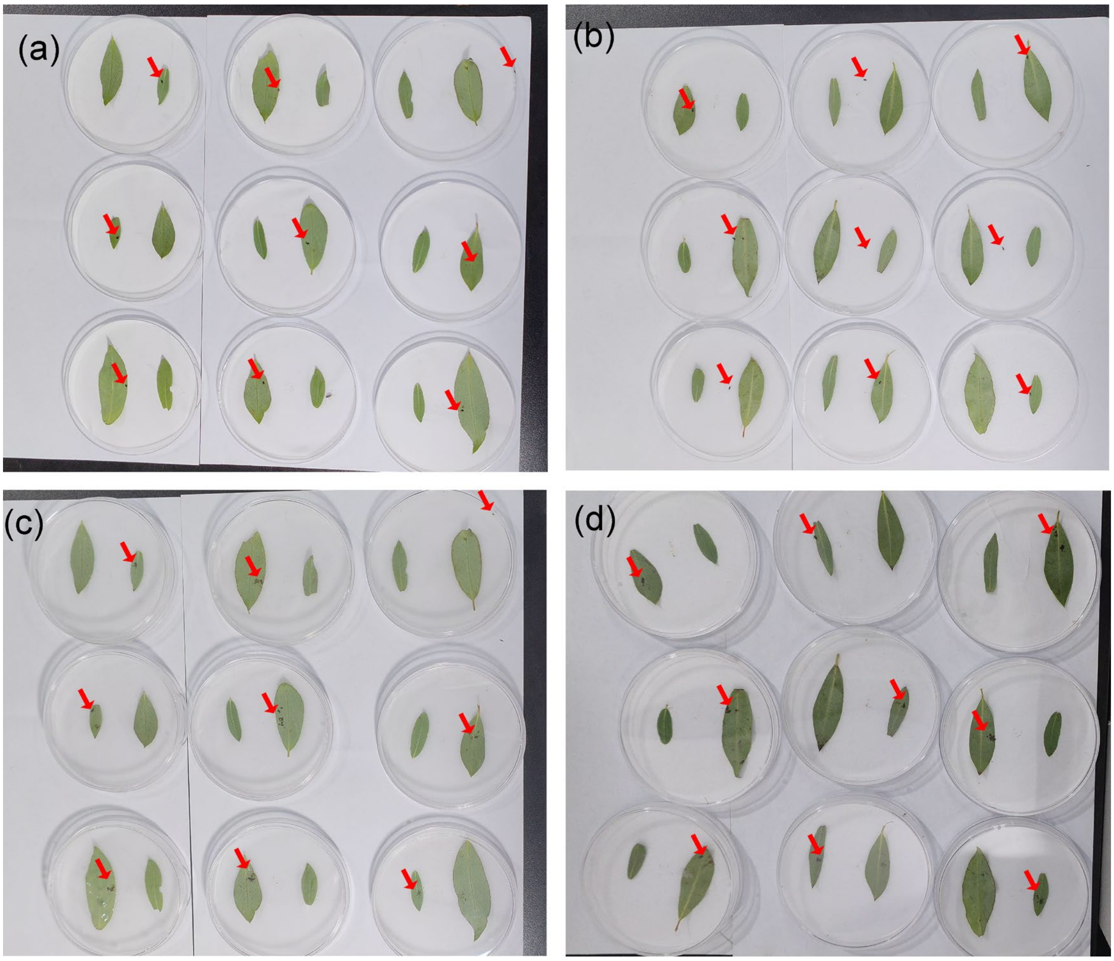

Figure S1. Representative snapshots of leaf size choice assays for CVS larvae, captured from continuous video recordings. Two independent replicates are shown for each time point: (a,b) at 1 h post-release, and (c,d) at 12 h post-release. Each dish contained one large and one small leaf disc, with red arrows indicating the larva's location.

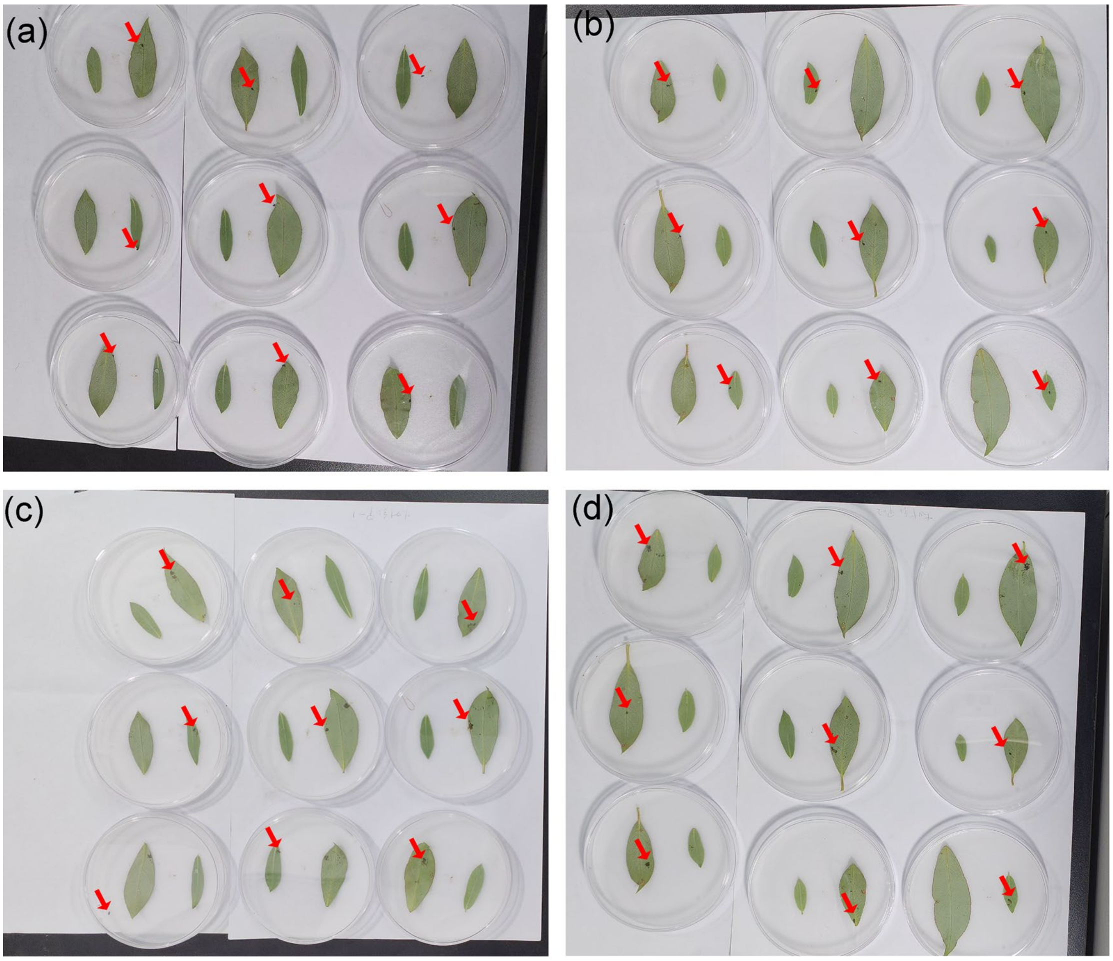

Figure S2. Representative snapshots of leaf size choice assays for CVL larvae, captured from continuous video recordings. Two independent replicates are shown for each time point: (a,b) at 1 h post-release, and (c,d) at 12 h post-release. Each dish contained one large and one small leaf disc, with red arrows indicating the larva's location.

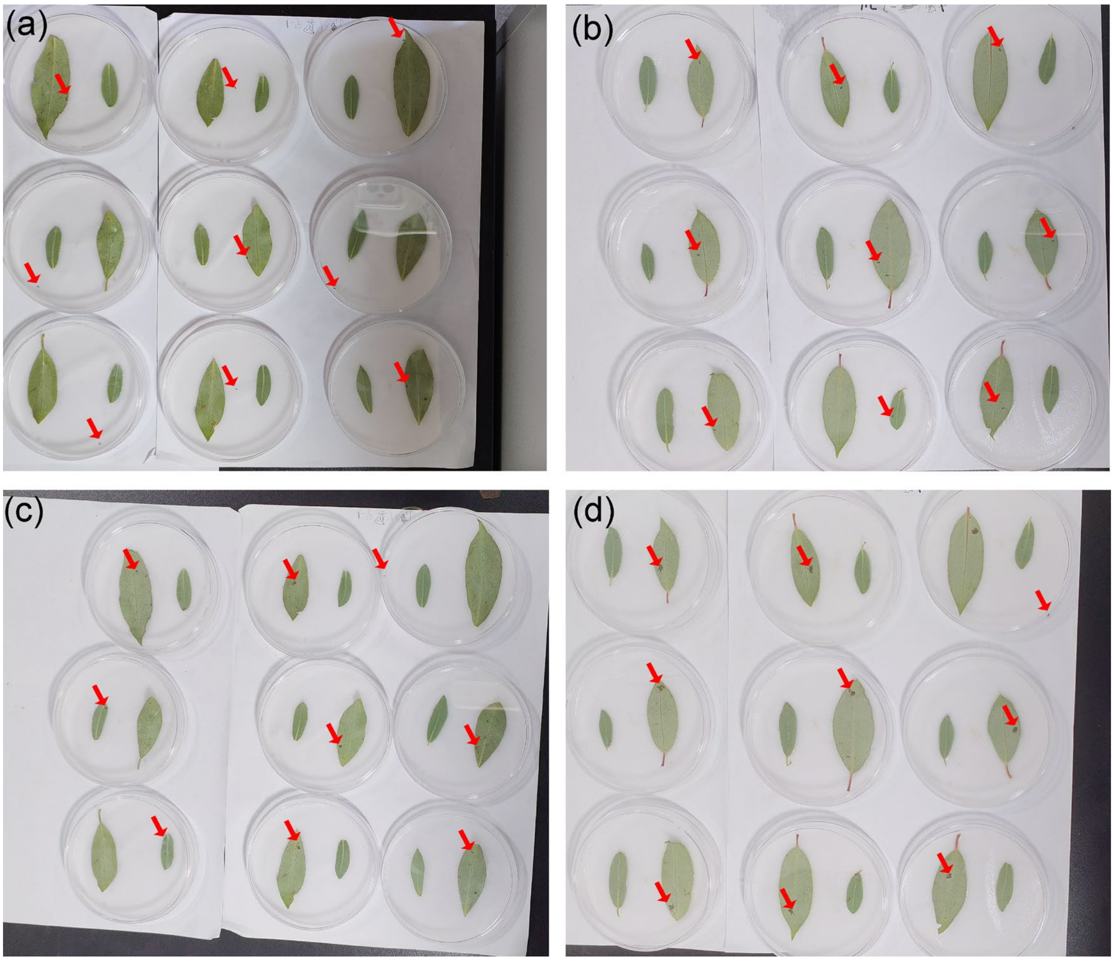

Figure S3. Representative snapshots of leaf size choice assays for PVS larvae, captured from continuous video recordings. Two independent replicates are shown for each time point: (a,b) at 1 h post-release, and (c,d) at 12 h post-release. Each dish contained one large and one small leaf disc, with red arrows indicating the larva's location.

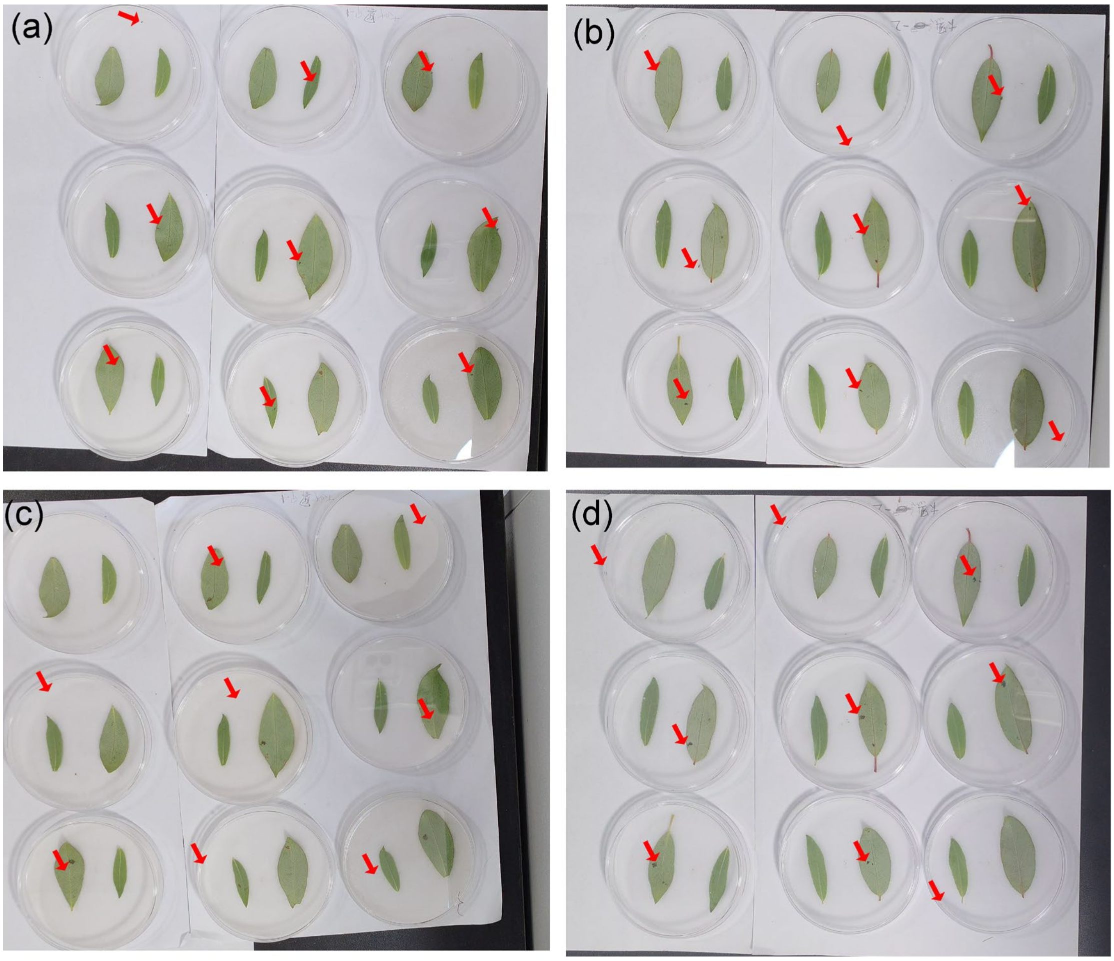

Figure S4. Representative snapshots of leaf size choice assays for PVL larvae, captured from continuous video recordings. Two independent replicates are shown for each time point: (a,b) at 1 h post-release, and (c,d) at 12 h post-release. Each dish contained one large and one small leaf disc, with red arrows indicating the larva's location. Dishes without an arrow in panel (c) indicate that the larva had escaped.

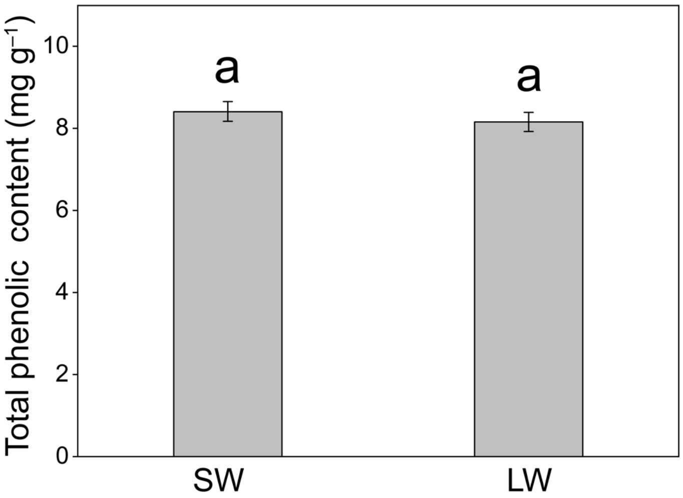

Figure S5. Total phenolic content between SW and LW. Different lowercase letters above bars denote statistically significant differences between the two willow species ($p < 0.05$).
